# The Quarantine Question

**Published:** 1898-11

**Authors:** 


### THE QUARANTINE QUESTION.

YELLOW fever has again appeared in the Southwest, just as it did one year ago. Considering the disrupted state of our country through the recent Spanish-American war, and the commingling together of the United States and those countries like Cuba and Porto Rico, where yellow fever is nearly always rampant, and considering also the severe epidemic we had last year and the very mild winter which has in the meantime intervened, we are not surprised at the present outbreak.

Mississippi, as usual, seems to be bearing the brunt of the epidemic. New Orleans has already been seriously affected, and commercial interests, due to quarantine, have also been jeopardized. Mississippi has appealed to the Federal Government for aid in the present epidemic, showing the inability of the authorities alone to cope with the disease. Such an appeal but forcibly represents the necessity, as we have always held, for a national quarantine control. Such a belief is in nowise a reflection upon the ability of State health officers, but it is a belief in the better extension of quarantine measures when under national control. State authorities, with their limited means, are unable to carry out any perfect system of quarantine, and when it is attempted there is frequently a clash between the health officers representing different communities of the same State. Yellow fever is demoralizing in many respects, and it is too serious a matter not to be dealt with in the most systematic manner. "Shotgun quarantine" is not the proper kind, but is even evil in its results. Of all the States in the South, none should be more thoroughly interested in the question of quarantine than Mississippi, and yet the officials from that State one year ago, in the convention assembled to discuss and devise plans for this very condition, opposed most strenuously the idea of national quarantine. Experience has certainly attested the fact that Federal control and regulations in quarantine matters is by far better than that executed by State authorities, and until we have a national quarantine the outlook must be gloomy. As one of our daily papers has well said, "there can be no question of States' rights in this matter, as is so much feared by strict constructionists. There can certainly be no more fear of the loss of States'

rights in controlling an epidemic than there is in bringing in national aid to repair its ravages after it has passed the danger point."

